# Investigation of Metallo(organo)siloxane—Polydimethylsiloxane Composites with a High Metallosiloxane Component Content

**DOI:** 10.3390/polym17223034

**Published:** 2025-11-16

**Authors:** Nadezhda A. Tebeneva, Alexander N. Tarasenkov, Ivan B. Meshkov, Aleksandra A. Kalinina, Alexander I. Buzin, Mikhail I. Buzin, Galina P. Goncharuk, Aziz M. Muzafarov

**Affiliations:** 1 N. S. Enikolopov Institute of Synthetic Polymer Materials, Russian Academy of Sciences (ISPM RAS), Profsoyuznaya 70, 117393 Moscow, Russia; tebeneva@mail.ru (N.A.T.); ivanbm@ispm.ru (I.B.M.); kalinina@ispm.ru (A.A.K.); buzinai@ispm.ru (A.I.B.); ggoncharuk@ispm.ru (G.P.G.); aziz@ispm.ru (A.M.M.); 2 A. N. Nesmeyanov Institute of Organoelement Compounds, Russian Academy of Sciences (INEOS RAS), Vavilova 28, 119991 Moscow, Russia; buzin@ineos.ac.ru

**Keywords:** metallosiloxane, polydimethylsiloxane, polycondensation, elastomer, silicon composition

## Abstract

A representative series of functional branched metallosiloxane oligomers was used to obtain polydimethylsiloxane-based composites highly filled with a metallosiloxane component. Physical and mechanical characteristics of compositions obtained strongly depends on metallosiloxane structure and composition. It is shown that it is possible to regulate the strength and elastic properties of the systems under consideration within wide limits, as well as to influence the morphology of the material. The resulting materials are rather thermo-oxidatively stable and can also maintain high mobility of polydimethylsiloxane chains.

## 1. Introduction

Development in the field of the synthesis of polymer materials with enhanced performance properties using original techniques for producing composites based on known polymer matrices remains an important scientific task. Filled silicone materials based on polydimethylsiloxane (PDMS) are currently among the most used in industrial, high-tech, and household applications [1,2].

Organosilicon fillers are most often used to achieve a high affinity of the matrix and the filler. One of the most common approaches is silica filling [3,4]. In addition to hardening [5,6], silica makes it possible to obtain superhydrophobic, antifouling materials and coatings [7,8,9,10,11], materials with improved fire resistance [12], and gas separation membranes [13], as well as various sealants and compounds [14,15,16,17,18]. At the same time, the physico-mechanical properties of the composite are greatly influenced by the factor of a good combination of silica with PDMS, including by means of its surface functionalization [19]. Also, filling with metal oxides nanoparticles (ZnO and TiO_2_) allows for the obtaining of silicone materials with good performance, electrical and thermal insulation, and optical, hydrophobic, gas separation, and antimicrobial properties and it can also be used to manufacture biocompatible and food materials [20,21,22,23,24].

A fundamentally different approach to filling silicones is their molecular filling. It consists of adding compounds to the initial composite that well disperse and combine with the matrix and undergo chemical transformations during the material formation, becoming a nanoscale filler. It may be organosoluble silica gels [25], as well as metallo(organo)siloxane (MS) oligomers, which high catalytic and filling activity in curing processes was previously shown in PDMS compositions [26,27]. Both variants are able to form, finally, a silica or organometal structure inside material during silica components condensation. In general, introduction of MS fillers into polymer matrices can lead to the production of highly heat-resistant conductive, magnetic, and optical materials with improved mechanical and dielectric characteristics [28,29,30,31]. Organosoluble MQ-copolymers are another promising siloxane filler for the production of PDMS nanocomposites [32]. In this case, the formation of a filled system from a homogeneous solution occurs only by removing the solvent. It has been shown that such a variant of filler generation can also be considered a molecular filling [33]. Molecular filling can solve one of the main problems in composite formation: ensuring uniform filler distribution in the final material. Furthermore, it can often improve compatibility between the material’s components.

The aim of this work is to screen functional MS oligomers of various structures and compositions in order to identify key structural elements that determine physico-mechanical and thermal characteristics of PDMS-based composites. Given the fact that computational methods for predicting the properties of nanocomposites are not available, and the filler is formed during the composite formation, the search algorithm is reduced to using the same initial matrices and a wide range of MS oligomers of different structures at the same concentrations of molecular filler. This approach will reveal the influence of both the filler concentration on the properties of the composite, as well as the structure of the MS oligomer and the chemical nature of its central metal atom.

## 2. Materials and Methods

The mechanical properties of the compositions were determined in the uniaxial extension mode on the universal testing machine “Autograph AGS-H” (Shimadzu, Kyoto, Japan). The samples were strips with a working part size of 3 × 20 mm; the rate of extension was 10 mm/min. The tensile strength and elongation at break were measured. The averaged data on three-five tests are presented.

Scanning electron microscopy (SEM) was performed using a JCM-6000 PLUS microscope (Tokyo, Japan) equipped with an energy-dispersive spectrometer at accelerating voltages of 5–15 kV. The samples were subjected to gold dusting before testing.

TGA analysis was performed on a “Derivatograph-C” device (Hungary). The measurement was carried out in air and argon atmosphere at a heating rate of 10 °C/min within the temperature range from 25 to 800 °C.

The DSC analysis was carried out on a “Perkin-Elmer DSC7” instrument (USA) at a heating rate of 20 °C/min in a nitrogen flow of 30 mL/min within the temperature range from −150 to 50 °C.

To prepare the compositions, commercially available Si-OH-terminated polydimethylsiloxane (PDMS) rubbers of different molecular weights were used as follows: “CKTH-A”—M_W_ ~ 20,000, 1.5–2 Pa·s; “CKTH-D”—M_W_ ~ 99,000; 18–25 Pa·s; and “CKTH-E”—M_W_ ~ 120,000, 80–120 Pa·s (hereinafter PDMS-A, -D, and -E, respectively).

MS oligomers were obtained according to the method [26,27,30,34].

PDMS-A* and -E* rubber pre-blocked with 3-aminopropyltriethoxysilane were obtained according to the procedure [20].

*Compositions preparation.* Toluene solution of initial components (PDMS rubber and MS) was poured onto a Teflon^®^ substrate and then kept at room temperature until most volatiles were evaporated. After that, residual was heated following the temperature steps: 1 h at 50 °C, then 1 h at 70 °C, then 1 h at 100 °C, and finally 2 h at 150 °C. The compositions were cooled, removed from the substrate, and then tested.

## 3. Results

### 3.1. Metallosiloxane Oligomers Synthesis

**Figure 1:**
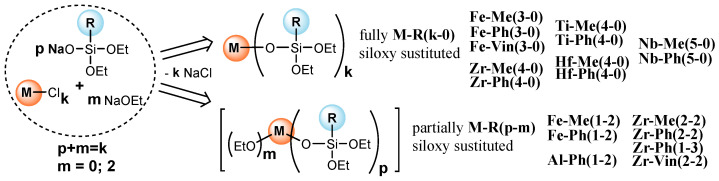
Metallosiloxanes used in this work.

A wide range of previously synthesized metallo(organo)ethoxysiloxane oligomers were used to obtain the compositions and study their physicochemical properties. They differed in the type of metal (Fe(III), Zr, Hf, Ti, and Nb), the degree of metal shielding by the siloxy substituent, and also the organosubstituent at the silicon atom (R = Me, Ph, Vin). The synthesis of such compounds was carried out by reacting the corresponding sodium organodiethoxysilanolate (and sodium ethoxide) with a metal chloride in an organic solvent medium, provided that the molar ratio [Cl]:[Na] = 1:1 n/n was maintained, yielding completely M[OSi(R)(OEt)2]k (M-R(k-0)) or partially (EtO)mM[OSi(R)(OEt)2]p (M-R(p-m)) (alkyldiethoxy)siloxy derivatives, where M is a metal atom, R is an organic group at silicon atom, k is the valence of the metal, and m is the degree of substitution of ethoxy groups at the metal atom (Figure 1). It was previously shown that such products are close in composition to the theoretical structures; however, they contain a small percentage of the oligomeric part [32,33]. When in contact with atmospheric moisture, these compounds easily condense to form three-dimensional networks containing covalently bonded metal atoms at the nodes, shielded by siloxane sections. Along with M-O-Si and Si-O-Si bonds, in the case of partially siloxy-substituted (alkyldiethoxy)siloxy derivatives, it is possible to form M-O-M bonds.

### 3.2. Obtaining the Composites

The main linear polymer for forming the composites was PDMS rubber of the “PDMS-D” brand. In this case, two components were involved in network forming of the final material: rubber and MS oligomer, where MS acts not only as a material-forming substance, but also as a catalyst due to the presence of an active metal center. To assess the effect of rubber on the quality of the formed material, PDMS rubbers of the “PDMS-A” and “PDMS-E” brands were also used.

**Figure 2:**
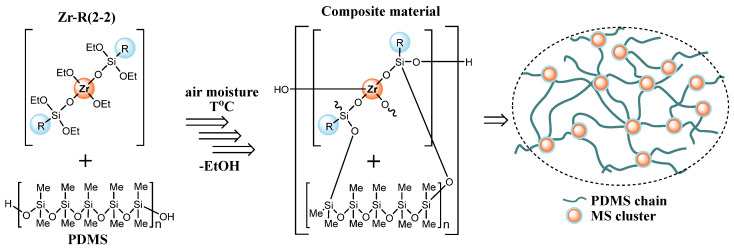
Scheme of two-component composite material formation.

In the presented study, the highly filled MS component composites were obtained. The initial mass fraction of MS relative to the {MS + PDMS} mixture was 0.5 wt.pt. Two parallel processes are assumed to occur when carried out in the open air with the participation of atmospheric moisture: binding of linear PDMS blocks with the MS and hydrolysis and condensation of MS with each other. The presence of an initially large excess of ethoxy groups of MS oligomer in the initial mixture relative to the silanol groups of rubber ([SiOEt]:[SiOH] ~ up to 500) causes the formation of two types of networks: organic, formed from PDMS, and metallosiloxane, formed by clusters of crosslinked MS (Figure 2). The compositions were obtained in the form of films with a thickness of 0.3–0.5 mm.

In fact, we obtained a nanocomposite (microcomposite in some cases) consisting of a PDMS chain network, the nodes of which contain MS clusters formed by the condensation of MS excess not bound to the rubber. These clusters will serve as the filler, and their size is determined not only by the MS amount introduced, but also by its structure and composition and, consequently, the dynamics of condensation processes and the compatibility of the final components of the system.

### 3.3. Mechanical Properties and Morphology of Composites

We have previously shown in various systems that an increase in the agent capable of forming a three-dimensional network upon curing leads to an increase in the mechanical tensile strength of the material, but at the same time, its elasticity decreases. In some cases, an increase in brittleness is observed with a very high filler content. The same can be expected from the systems under consideration. Table 1, Table 2 and Table 3 show the mechanical characteristics of the composites.

The data obtained show that when using PDMS-D rubber, only in some cases (using Ph-derivatives of MS) can an elastomeric homogeneous material be obtained with an initial mass fraction ω(MS) = 0.5 wt.pt., while in most cases, a strong inelastic material is formed. In this case, the calculated content of the formed MS filler in the cured material is in the range of 35–40% wt., depending on the type of MS.

In general, Fe- and Zr-containing compositions have similar tendencies in the influence of the hardener on the PDMS-D-based composites’ mechanical properties (Table 1 and Table 2). With an initial mass fraction ω(MS) = 0.5–0.6 wt.pt. in the mixture, the tensile strength (σ) of the composites varies in the range of 1.4–10.0 MPa. At the same time, the use of Me-derivatives of MS results in compositions that are stronger in tensile strength, but less in elastic than the use of Ph-derivatives of MS. Thus, the strength of the composites with the maximum content of the fully Me–siloxy-substituted MS component varies within 5.8–9.2 MPa (with a relative deformation (ε) of no more than 20%) as opposed to 1.4–3.3 MPa (ε up to 400%) with the fully Ph–siloxy-substituted MS component. This behavior in the case of Ph-derivatives can be associated with partial shielding of ethoxy groups of MS and, as a consequence, the formation of a noticeable share of a more elastic PDMS network and a decrease in the strengthening role of MS clusters due to their less uniform incorporation into the PDMS network. The same was observed earlier for three-component compositions {MS + PDMS + polyethoxysiloxane} [28]. The nature of the compositions when using Vin-derivatives Fe-Vin(3-0) and Zr-Vin(2-2) is similar to ones at the use of Me-derivatives. Indeed, the strengthening effect is illustratively confirmed by the highest values of Young’s modulus during curing with completely Me–siloxy-substituted MS: E_0_ ~ 46–200 MPa; however, the increase in the E_0_ correlates with a significant decrease in the elasticity of the compositions. Conversely, E_0_ ~ 0.3–5 MPa during curing with completely Ph–siloxy-substituted MS, which are more elastic.

**Figure 3:**
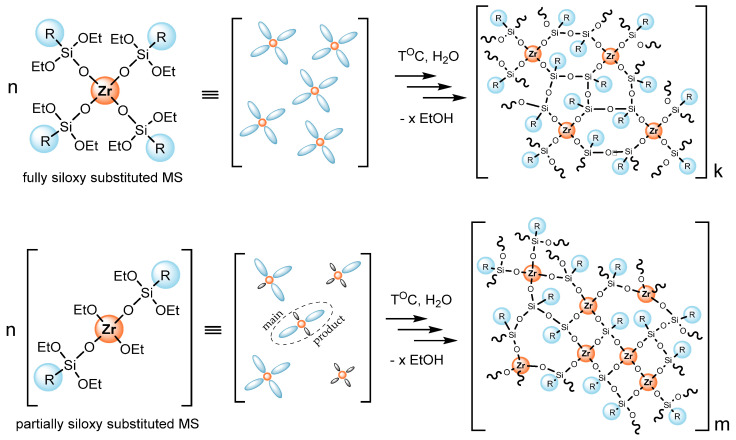
Scheme of MS network formation in example of Zr–siloxanes differing in metal atom shielding.

When using partially siloxy-substituted MS, the above-described dependencies are preserved. However, higher values of the compositions’ strength in the case of partially Ph–siloxy-substituted MS compared to fully Ph–siloxy-substituted analogs are noteworthy. Thus, the initial system Fe-Ph(1-2):PDMS-D = 0.6:0.4 wt.pt. after curing gives a composition with a 10.3 MPa tensile strength, which is comparable to aerosil-filled PDMS. Such effect can be explained by two factors. Firstly, the presence of M-OEt groups causes a higher reactivity with respect to hydrolysis by atmospheric moisture, and, as a consequence, more confident formation of MS clusters including self-condensation of MS excess. Secondly, assuming the averaged composition of partially siloxy-substituted MS, it may contain a certain proportion of structures with a greater or lesser content of M-O-Si and M-OEt groups and potentially be capable of forming clusters of non-uniform density, including denser and more rigid networks, due to the appearance of M-O-M and a decrease in the proportion of Si-O-Si fragments (Figure 3).

Metal atom changing in the MS oligomer structure effects the kinetics of the material network formation (Table 3). When examining fully siloxy-substituted Ti– and Hf–siloxanes, it is evident that the containing compositions differ from Zr-containing analogues in their properties. Thus, the presumably higher reactivity of Ti-Me(4-0) does not allow obtaining elastomer compositions with an initial ω(MS) = 0.5 wt.pt., and the use of Ti-Ph(4-0) does not result in obtaining a homogeneous material. The kinetics factor is also evident in the example of using Nb-siloxanes. Capable of theoretically forming the most densely crosslinked network, Nb-Me(5-0) produces a more elastic material than Fe-Me(3-0) under the same conditions, while its Ph-containing analogue Nb-Ph(5-0) produces brittle samples of curable silanol rubber.

**Figure 4:**
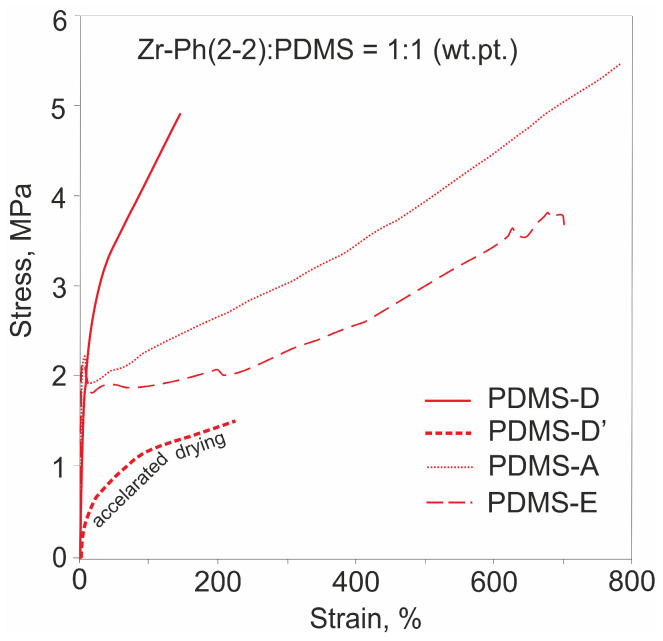
Examples of tensile curves when using different curing approaches.

As shown earlier, it is possible to influence the mechanical properties of PDMS composites cured with MS by changing the drying rate of the initial mixture and the length of the PDMS rubber, thereby changing the initial [OEt]:[Si-OH] ratio in the cured system [28]. The systems considered here are no exception. Both options are illustrated for cases of using different MS (Table 1, Table 2 and Table 3). Thus, keeping more concentrated and, as a result, faster drying initial mixtures in air generally allows for the obtaining of more elastic composites with a slightly reduced tensile strength (shown using Fe-Me(3-0), Nb-Me(5-0), and Zr-Ph(2-2) as an example). However, this can also worsen the compatibility of the system final components and the mechanical properties, as in the case of Zr-Vin(2-2). In most of the cases considered, changing the number of silanol groups in the system to a greater (when using PDMS-A) or lesser (PDMS-E) amount allows for the increasing of the elasticity of the compositions both by reducing the proportion of MS clusters in the first case and by increasing the PDMS chain length between the network nodes in the second. In this case, there is a high probability of reducing the proportion of elastic deformations and increasing irreversible deformations with the formation of a “neck”. The presence of a “neck” can be explained by the formation of sufficiently large MS clusters in the material structure and is more typical for cases of using M-Me(x-0) and M-R(x-2). The use of PDMS-A* rubber, pre-blocked with 3-aminopropyltriethoxysilane at the chain end, allows for the excluding of the silanol component from the initial system, thereby leveling the rate of ethoxy group condensation processes and increasing the elasticity of the system due to crosslinking of individual rubber chains (shown using Fe-Me(3-0) and Hf-Ph(4-0) as an example). In the case of the use of Nb-Ph(5-0), this turned out to be the only way to form an elastomer composition, with an initial ω(MS) up to 0.7 wt.pt. relative to the entire mixture, which corresponds to 60% wt. of the filler. The mechanisms considered are intended to influence the kinetics of formation and the ratio of MS and PDMS networks in the system, which in turn can affect the homogeneity of the final material and its mechanical characteristics (Figure 4).

**Figure 5:**
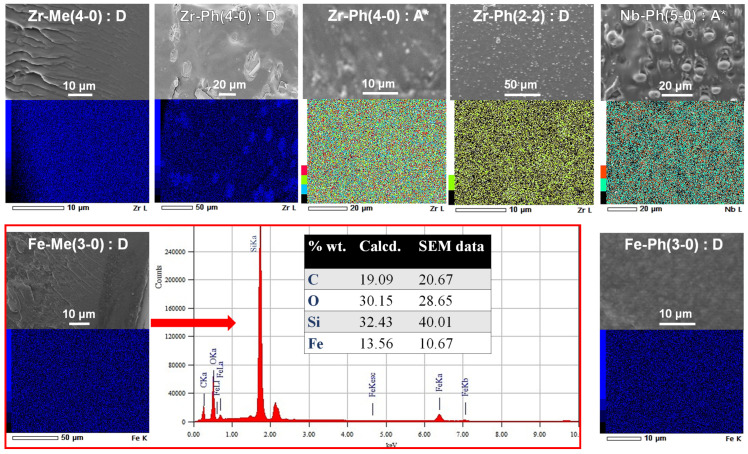
SEM data of the composites obtained from the MS:PDMS = 1:1 wt.pt. initial system.

**Figure 6:**
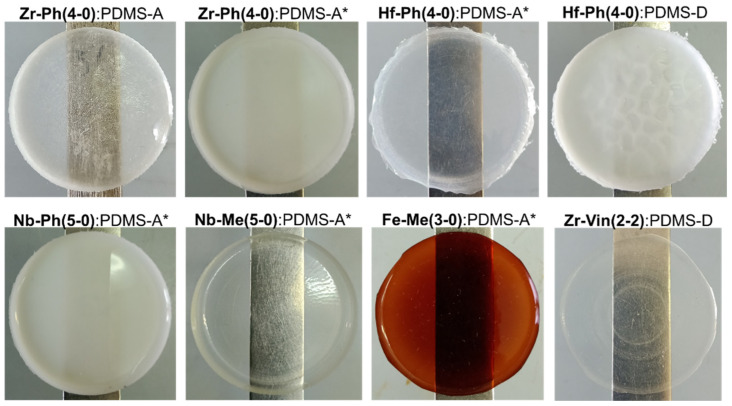
View of some samples obtained from the MS:PDMS = 1:1 wt.pt. initial system (* PDMS pre-blocked with 3-aminopropyltriethoxysilane).

The SEM study of a number of samples revealed that microhomogeneity is more characteristic of them in the case of the use of Me–siloxy-substituted MS (Figure 5). The case of the use of Ph–siloxy-substituted MS often produces the microcomposite formation as a result. Thus, in the case of using Zr-Ph(4-0), obvious phase segregation is observed with the formation of spherical elongated 20–30 μm domains, enriched with zirconium. At the same time, in the case of Zr-Ph(2-2), 3–5 μm particles are formed. The use of pre-blocked rubber PDMS-A* can contribute to a decrease in the phase-separated particles size in these cases. However, in the case of Nb-Ph(5-0) and PDMS-A* used together, the formation of up to 7 μm particles is observed, which is facilitated by the tendency to form its own denser MS network. Samples with a microcomposite nature are characterized by external opalescence or turbidity (Figure 6). Elemental maps in almost all cases show a consistent metallosiloxane component distribution throughout the material, further demonstrating the advantage of the chosen silicone material filling method. It should also be noted that energy-dispersive X-ray microanalysis shows the elemental content in the material to be close to the calculated one, indicating the absence of any destructive processes under the temperature conditions of material formation.

### 3.4. Thermophysical Properties of Composites

Thermophysical properties of composites were studied using samples of the initial composition MS:PDMS = 1:1 wt.pt. (Table 4).

**Figure 7:**
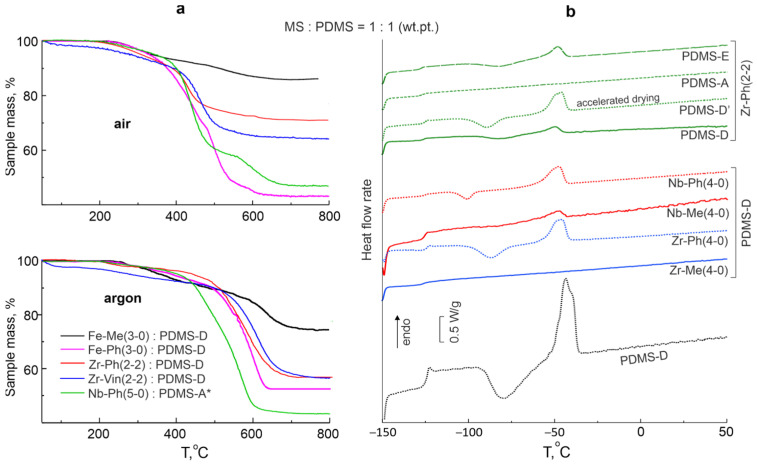
Examples of TGA (**a**) and DSC (**b**) thermograms of compositions obtained from the initial MS:PDMS = 1:1 wt.pt. system (* PDMS pre-blocked with 3-aminopropyltriethoxysilane).

For composites obtained using fully siloxy-substituted MS, the losses of 5% wt. of the initial mass are in the range of T_5%_ = 290–365 and 270–430 °C, and for partially siloxy-substituted 270–315 and 260–450 °C in air and in argon, respectively. The least air thermally stable are composites obtained using Fe-Me(1-2) and Hf-Ph(4-0). This illustrates the influence of the MS oligomer structure (organic group type/catalytic properties of metal atom) on the thermal stability of composites (Figure 7a). Nevertheless, we can talk about the rather high thermal and thermal-oxidative stability of the composites. When considering a 10% mass loss, thermal stability of the materials increases significantly. Coke residues vary in a wide range of 42–86% wt. and are also determined by MS structure and composition.

The glass transition temperatures T_g_ are in the range from −124 to −127 °C, which corresponds to PDMS rubber (−124 °C). However, some of the compositions can be characterized as amorphous–crystalline, since they have a melting region on the DSC thermograms in the range of Tm = −45−53 °C (Figure 7b), which corresponds to the melting temperature of pure PDMS rubber (−44 °C). This fact indicates the preservation of the mobility of PDMS chains between the network nodes inside such a material, provided that PDMS-D rubber is used even with a high filling of the MS component. The effect is characteristic of all samples containing Ph– and Vin–siloxy-substituted MS components, and is not characteristic in the presence of Me–siloxy-substituted MS components, except for the cases of Fe-Me(3-0) and Nb-Me(5-0). This behavior is consistent with the hypothesis that in the case of using Ph-derivatives a slight segregation between MS clusters and PDMS networks is observed, and PDMS network retains mobility. The use of lower-molecular-weight PDMS-A rubber leads to a decrease in the chain length between the network nodes and, as a result, “melting region” disappears from the thermograms in almost all cases, except for the Zr-Ph(4-0) case. Conversely, when using higher-molecular-weight PDMS-E rubber, the “melting region” not only does not disappear, but its thermal effect increases, except for the Zr-Vin(2-2) case. The use of pre-blocked PDMS-A* rubber does not have a clear effect on the presence/absence of “melting region” on the thermograms; in this case, the phase behavior is determined by the MS structure and composition. Faster drying of the original system also has virtually no effect on the phase behavior of the composite.

## 4. Conclusions

A representative series of functional branched metallosiloxane oligomers differing in the type of metal, structure, and type of substituent at the silicon atom was used to obtain polydimethylsiloxane composites highly filled with a metallosiloxane component. The study of their physical and mechanical characteristics showed that the strength of the compositions increases and elasticity decreases with an increase in the metallosiloxane content. At the same time, metallosiloxane structure and composition play a decisive role in regulating the properties of the resulting composites, depending on which both highly filled elastic amorphous–crystalline composites and rigid materials with strength comparable to polydimethylsiloxane rubber filled with aerosil can be obtained. The materials under consideration have a rather high thermal-oxidative stability (300–400 °C) and can also maintain high mobility of polydimethylsiloxane chains within the material. The resulting compositions can potentially find application as sealants, protective coatings with adjustable elasticity/rigidity, high heat resistance, and resistance to solvents.

## Figures and Tables

**Table 1:** Mechanical properties of samples of the initial Fe-R:PDMS = x:(1–x) wt.pt. composition.

#	MS	PDMS	x, wt.pt.	ω, % wt.	$\frac{\sigma \pm \Delta \sigma }{\varepsilon \pm \Delta \varepsilon }$ MPa/%	E_o_ ± ΔE_o_, MPa	Characteristics
1	Fe-Me(3-0)	D	0.5	36	6.0 ± 0.8 7 ± 1	161.9 ± 8.9	Dark brown, transparent, homogeneous
2		D ^c^		36	5.2 ± 0.3 605 ± 73 *^b^*	74.4 ± 2.9	Dark brown, transparent, homogeneous, “grain” of the surface
3		A *		35	4.6 ± 0.4 296 ± 86 *^b^*	71.6 ± 6.5	Dark brown, transparent, homogeneous
4	Fe-Ph(3-0)	D	0.5	40	2.1 ± 0.1 138 ± 27	5.3 ± 0.7	Dark brown, opaque, uniform
5	Fe-Vin(3-0)	D	0.5	37	4.5 ± 0.8 3 ± 1	206.5 ± 10.9	Dark brown, transparent, homogeneous
6	Fe-Me(1-2)	D	0.5	39	5.9 ± 1.3 4 ± 1	261.7 ± 25.6	Dark brown, opaque, uniform
7	Fe-Ph(1-2)	D	0.5	37	5.8 ± 0.6 152 ± 64	125.2 ± 10.1	
8		D	0.6	47	10.3 ± 0.3 12 ± 2 *^a^*		

x—mass fraction of MS oligomer in initial system; ω—calculated mass fraction of MS filler in the cured material; ^*a*^—rupture during neck formation; ^*b*^—rupture during neck propagation; ^c^—more concentrated initial system; * PDMS pre-blocked with 3-aminopropyltriethoxysilane. Young’s modulus was measured only for compositions with an initial fraction of MC x = 1.

**Table 2:** Mechanical properties of samples of the initial Zr-R:PDMS = x:(1–x) wt.pt. composition.

#	MS	PDMS	x, wt.pt.	ω, % wt.	$\frac{\sigma \pm \Delta \sigma }{\varepsilon \pm \Delta \varepsilon }$ MPa/%	E_o_ ± ΔE_o_, MPa	Characteristics
1	Zr-Me(4-0)	D	0.5	36	6.3 ± 0.1 16 ± 1 *^a^*	135.4 ± 8.1	Colorless, transparent, homogeneous
2			0.6	46	6.9 ± 0.7 7 ± 1		
3	Zr-Ph(4-0)	D	0.5	41	1.4 ± 0.1 458 ± 54	0.3 ± 0.03	Yellowish, opalescent, heterogeneous morphology
4		A		40	2.1 ± 0.4 222 ± 55	1.0 ± 0.01	
5		E		41	-	-	Yellowish, inhomogeneous, cracked surface
6		A *		40	2.6 ± 0.3 256 ± 63	1.5 ± 0.1	White, homogeneous
7	Zr-Me(2-2)	D	0.5	35	3.2 ± 0.5 4 ± 1	117.2 ± 6.8	Colorless, transparent, homogeneous
8	Zr-Ph(2-2)	D	0.5	39	4.9 ± 0.4 140 ± 31	56.3 ± 3.3	
9		D ^c^		39	1.4 ± 0.1 209 ± 15	1.7 ± 0.1	Whitish, cloudy, homogeneous
10		A		38	5.2 ± 0.4 820 ± 71 *^b^*	101.9 ± 2.8	Colorless, transparent, homogeneous
11		E		39	3.6 ± 0.2 637 ± 61 *^b^*	77.9 ± 6.4	Colorless, opalescent, homogeneous
12		D	0.6	49	7.4 ± 0.6 14 ± 6		Colorless, transparent, homogeneous
13	Zr-Ph(1-3)	D	0.5	37	4.5 ± 0.3 7 ± 1 *^a^*	138.2 ± 4.2	
14	Zr-Vin(2-2)	D	0.5	36	4.7 ± 0.1 280 ± 49 *^b^*	102.6 ± 2.5	Yellowish, transparent, homogeneous
15		D ^c^		36	2.8 ± 0.1 19 ± 7 *^b^*	80.3 ± 1.9	Yellowish, transparent, homogeneous, “grain” of the surface
16		A		36	4.3 ± 0.1 184 ± 22 *^b^*	85.4 ± 3.5	Yellowish, transparent, homogeneous
17		E		36	4.8 ± 0.1 114 ± 31 *^b^*	124.4 ± 6.4	

x—mass fraction of MS oligomer in initial system; ω—calculated mass fraction of MS filler in the cured material; ^*a*^—rupture during neck formation; ^*b*^—rupture during neck propagation; ^c^—more concentrated initial system; * PDMS pre-blocked with 3-aminopropyltriethoxysilane. Young’s modulus was measured only for compositions with an initial fraction of MC x = 1.

**Table 3:** Mechanical properties of samples of the initial M-R(k-0):PDMS = x:(1–x) wt.pt. composition.

#	MS	PDMS	x, wt.pt.	ω, % wt.	$\frac{\sigma \pm \Delta \sigma }{\varepsilon \pm \Delta \varepsilon }$ MPa/%	E_o_ ± ΔE_o_, MPa	Characteristics
1	Ti-Me(4-0)	D	0.5	35	1.5 ± 0.1 1 ± 1	187.2 ± 16.9	Colorless, transparent, homogeneous
2	Ti-Ph(4-0)	D	0.5	40			
3		D ^c^		40			Yellowish, opalescent, heterogeneous morphology
4		A *		38	3.2 ± 0.2 260 ± 71	3.8 ± 0.1	
5	Hf-Me(4-0)	D	0.5	38	5.8 ± 0.3 22 ± 2 *^a^*	101.0 ± 2.8	Yellowish, inhomogeneous, cracked surface
6	Hf-Ph(4-0)	D	0.5	41	3.3 ± 0.2 421 ± 25	3.0 ± 0.4	White, homogeneous
7		A *		41	5.9 ± 1.6 610 ± 179	4.5 ± 0.8	Colorless, transparent, homogeneous
8	Nb-Me(5-0)	D	0.5	36	9.2 ± 0.5 20 ± 3	87.1 ± 2.7	
9		D ^c^		36	4.6 ± 0.1 80 ± 18 *^b^*	83.6 ± 3.4	Whitish, cloudy, homogeneous
10		A		36	5.9 ± 0.1 73 ± 4	46.5 ± 1.8	Colorless, transparent, homogeneous
11		A *		35	4.8 ± 0.3 19 ± 2	62.3 ± 1.4	Colorless, opalescent, homogeneous
12		E		36	5.0 ± 0.5 573 ± 103 *^b^*	57.9 ± 0.3	Colorless, transparent, homogeneous
13		D	0.6	46	5.0 ± 1.4 3 ± 1		
14	Nb-Ph(5-0)	D	0.5	40			Colorless, transparent, homogeneous, fragile
15		A *		40	2.5 ± 0.2 249 ± 18	0.7 ± 0.1	Yellowish, transparent, homogeneous, “grain” of the surface
16		A *	0.6	50	2.4 ± 0.1 320 ± 44		Yellowish, transparent, homogeneous
17		A *	0.7	61	1.9 ± 0.1 85 ± 13		

x—mass fraction of MS oligomer in initial system; ω—calculated mass fraction of MS filler in the cured material; ^*a*^—rupture during neck formation; ^*b*^—rupture during neck propagation; ^c^—more concentrated initial system; * PDMS pre-blocked with 3-aminopropyltriethoxysilane. Young’s modulus was measured only for compositions with an initial fraction of MC x = 1.

**Table 4:** Thermal characteristics of samples of the initial Zr-R:PDMS = 1:1 wt.pt. composition.

#	MS	PDMS	TGA						DSC		
			T_5%_, °C		T_10%_, °C		Coke, % wt.		T_g_, °C	T_m_, °C	ΔH_m_, J/g
			Air	Argon	Air	Argon	Air	Argon			
1	Fe-Me(3-0)	D	343	386	531	484	86	75	−127	−51	1
2		D *^a^*							−126	−49	8
3		A *							−125	-	-
4	Fe-Me(1-2)	D	272	272	337	356	77	73	−126	-	-
5	Fe-Ph(3-0)	D	329	430	385	535	68	57	−125	−45	13
6	Fe-Ph(1-2)	D	315	338	363	485	68	53	−125	−51	2
7	Zr-Me(4-0)	D	330	390	374	438	75	43	−126	-	-
8	Zr-Ph(4-0)	D	301	343	369	444	54	47	−124	−46	13
9		A							−126	−50	3
10		A *							−125	−50	10
11	Zr-Me(2-2)	D	315	352	374	380	79	53	−125	-	-
12	Zr-Ph(2-2)	D	315	446	401	523	74	61	−126	−49	4
13		D *^a^*							−125	−46	11
14		A							−126	-	-
15		E							−127	−48	6
16	Zr-Vin(2-2)	D	310	258	405	485	65	56	−126	−53	1
17		D *^a^*							−125	-	-
18		A							−124	-	-
19		E							−125	-	-
20	Ti-Me(4-0)	D	325	420	446	445	82	56	−126	-	-
21	Ti-Ph(4-0)	A *	330	352	371	404	55	57	−125	−50	9
22	Hf-Me(4-0)	D	357	398	406	446	79	50	−127	-	-
23	Hf-Ph(4-0)	D	292	273	432	468	57	46	−125	−46	11
24		A *							−124	−52	<0.5
25	Nb-Me(5-0)	D	365	418	439	450	85	65	−125	−49	2
26		D *^a^*							−125	−49	2
27		A							−124	-	-
28		A *							−124	-	-
29		E							−125	−50	4
30	Nb-Ph(5-0)	A *	325	382	394	445	45	42	−125	−48	12

*^a^*—more concentrated initial system; * PDMS pre-blocked with 3-aminopropyltriethoxysilane; T_m_/ΔH_m_—melting temperature and thermal effect. TGA data was determined mostly only for PDMS-D containing compositions.

## Data Availability

The original contributions presented in this study are included in the article. Further inquiries can be directed to the corresponding author.
